# New findings of dermoscopy and reflectance confocal microscopy for perforating pseudoxanthoma elasticum with elastosis perforans serpiginosa-like changes

**DOI:** 10.1016/j.jdcr.2024.09.031

**Published:** 2024-11-26

**Authors:** Juan Zhao, Ruiya Li, Hongyong Sun, Yan Duan

**Affiliations:** Department of Dermatology, People's Hospital of Inner Mongolia Autonomous Region, Hohhot, Inner Mongolia, China

**Keywords:** dermatoscopy, elastosis perforans serpiginosa, pseudoxanthoma elasticum, reflectance confocal microscopy

*To the Editor:* Pseudoxanthoma elasticum (PXE) and elastosis perforans serpiginosa (EPS).are both connective tissue disorders. The former is characterized by the calcification of elastic fibers due to a mutation in the ABCC6 gene, and associated systemic findings mainly include cardiac and ophthalmologic alterations, which can lead to life-threatening complications. The latter is considered as a mild dermatosis that is not usually accompanied by involvement of other organs, characterized by the transepidermal elimination of altered elastic tissue. Therefore, it is important to differentiate between PXE and EPS. PXE, which has sometimes been observed in.association with EPS-like changes, resulting from the transepidermal elimination.of altered elastic tissue—a condition that has been defined as either EPS-like PXE or perforating PXE.^1^, ^2^, ^3^ In this case, we described some new findings of dermoscopy and reflectance confocal microscopy (RCM) for EPS-like PXE.

A 63-year-old man affected yellow and red plaques on his neck 10 years ago, and brown plaques gradually appeared on his back and buttocks. It was found that during a physical examination, brown keratinized plaques with atrophic scars and pigmentation were distributed on the back and buttocks (Fig 1, *A*). Yellowish-red plaque, keratotic, centrally depressed plaque with sharply defined and slightly raised edges of 2 by 2.5 cm in diameter were detected on the middle of the neck, .there were many strawberry seed-like particles in the center of the plaque (Fig 1, *B*, *yellow arrow*). Two yellowish, waxy, and hyperextensible plaques were found on the left posterolateral side of the neck (Fig 1, *B*, *green arrow*). The patient denied a family history of PXE and any history of drug intake. No obvious abnormalities were found during the physical examination of the heart, lungs, and abdomen.

**Fig 1:**
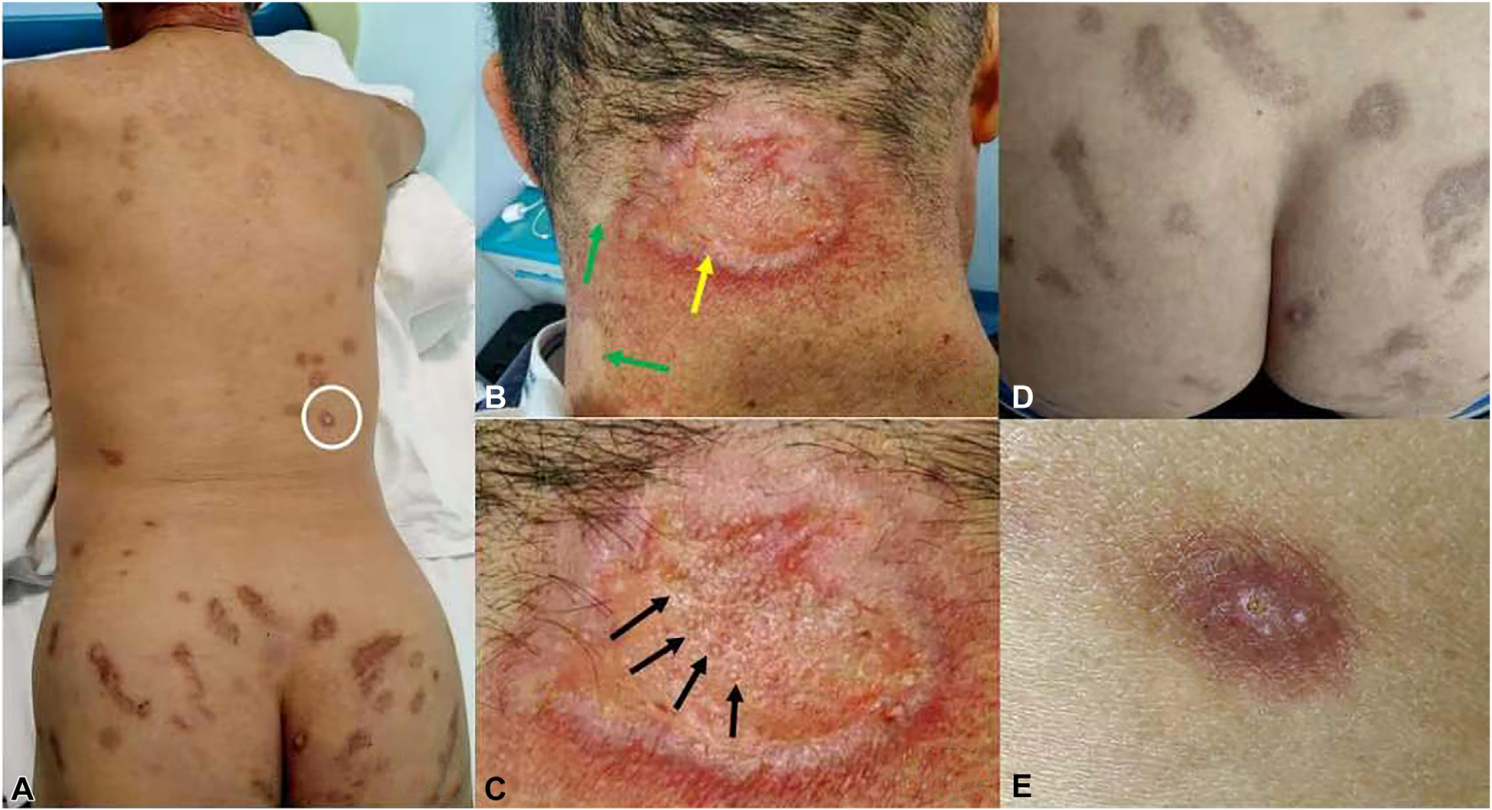
The patient’s clinical pictures. **A,** Skin lesions on the back and buttocks, *brown* keratinized plaques, multiple atrophic scars, and *brown* pigmentation. **B,** Skin lesions on the neck. A *yellowish*, waxy, and hyperextensible plaque (*green arrow*). *Yellowish* to reddish keratotic plaque on the neck (*yellow arrow*). **C,** Magnificated image of the lesion indicated by the *yellow arrow* in (**B**), showing a centrally depressed plaque with strawberry seed-like white particles in the center (*black arrow*) and sharply defined, slightly raised edges. **D,** Skin lesions on the buttocks, multiple *brown* atrophic scars, and *brown* pigmentation. **E,** Magnificated image of the *white circle* in (**A**) showing a *reddish-brown* keratinized plaque with *white particles* on the surface.

The polarized light dermoscopy findings (Fig 2) for the yellowish to reddish plaque (Fig 1, *B*, *yellow arrow*) showed yellowish-orange color alternating with reddish and whitish areas, some microulcerations (Fig 2, *A*, *purple arrow*), multiple white keratotic plugs like “strawberry seeds” (Fig 2, *A*, *green arrow*) on an erythematous background. The dermoscopy findings of the brown plaque on the back (Fig 1, *D*) showed central white keratotic plugs.(Fig 2, *B*, *red arrow*), peripheral white scar-like stripes, and brown pigmentation (Fig 2, *B*, *blue arrow*). The features of RCM were shown in Fig 3. The RCM images revealed well-defined, oval, moderate to hyperreflective lumpy material on the epidermis (Fig 3, *A*). The dermo-.epidermal junction was disarranged with hyperreflective material filling the dermal papillae and even reaching the skin surface (Fig 3, *B*). Hyperreflective curly amorphous material in the upper dermis resembled “cotton wadding” (Fig 3, *C*). The absence of epidermis and scattered inflammatory cell infiltration were observed in the dermal papillae (Fig 3, *D*).The findings of the 2 devices on the neck and back are similar, but microulcerations were only found on the neck. Histopathologic examination (Fig 4) and Elastic-Van Gieson staining (Fig 5) confirmed the diagnosis of EPS-like PXE.

**Fig 2:**
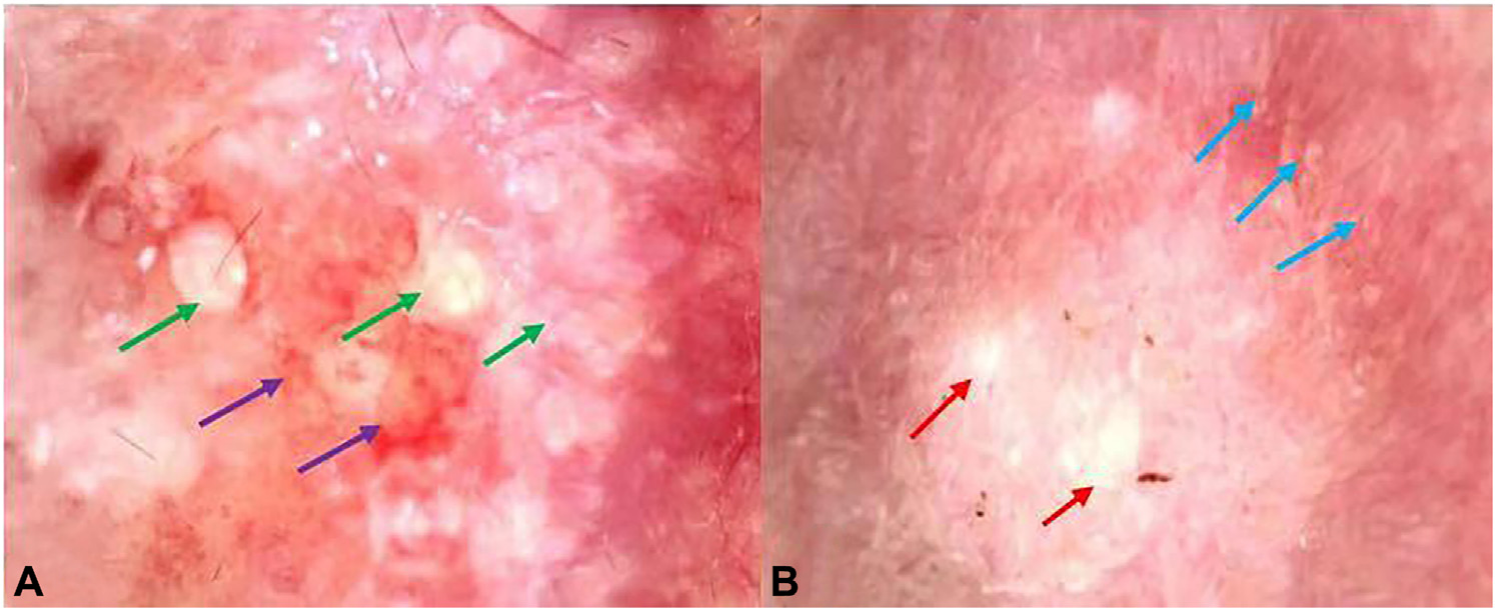
The dermoscopy findings for lesions on the neck and back ( ×10 polarized light). **A,** The dermoscopy image of the lesion on the neck (Fig 1, *B green arrow*): *Yellowish-orange* color alternating with *reddish* and *whitish* areas, some microulcerations (*purple arrow*), keratotic plugs (*green arrow*) on an erythematous background. **B,** The dermoscopy image of the lesion on the back (Fig 1, *E*) central white corner plug (*red arrow*), peripheral *white* scar like stripes and *brown* pigmentation (*blue arrow*).

**Fig 3:**
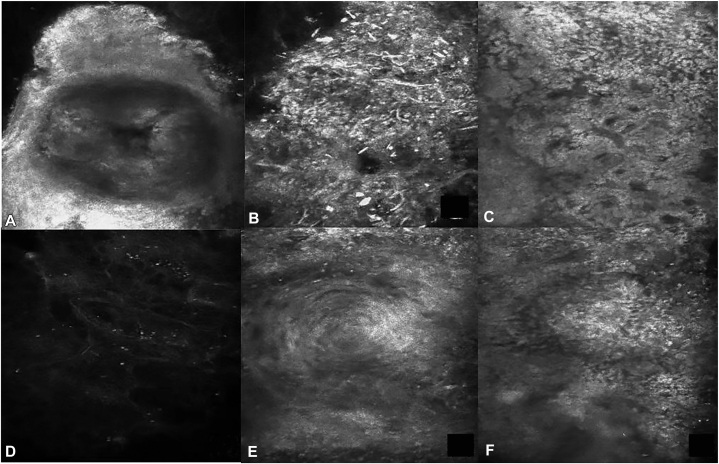
The RCM features of lesions on the neck and back. **A**-**D,** are the images of the lesion on the neck (Fig 1, *B yellow arrow*). **A,** Well defined, oval, moderate to hyperreflective lumpy material on the epidermis. **B,** Curled, frayed, elastic fibers reached the skin surface. **C,** Hyperreflective curly amorphous material on the upper dermis just like “cotton wadding”. **D,** Absence of epidermis and scattered inflammatory cell infiltration on the upper dermis. **E** and **F,** are the images of the lesion on the back (Fig 1, *E*). **E,** A moderate to hyperreflective oval, whirlpool like area on the epidermis. **F,** Hyperreflective amorphous material on the dermal papillae. *RCM*, Reflectance confocal microscopy.

**Fig 4:**
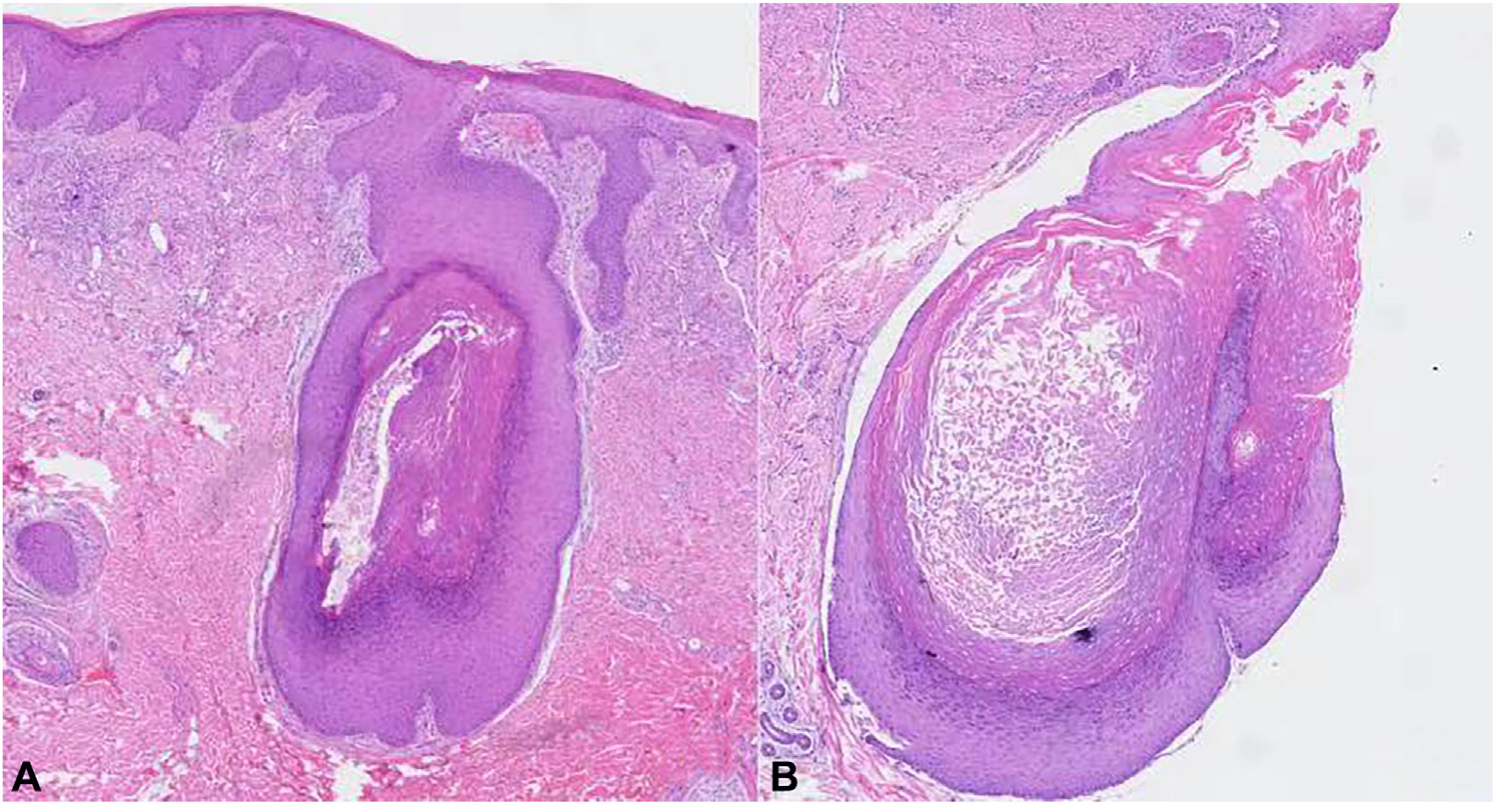
Histopathology of the lesion on the neck (Fig 1, *B yellow arrow*) (HE × 40). **A,** Moderately acanthotic epidermis, altered elastic fibers, and calcified material are wrapped by the epidermis, numerous fragmented, stringy, and curled elastic fibers giving the appearance of raveled wool in the upper-and mid-reticular dermis. **B,** A transepidermal perforating channel containing elastic fibers and basophilic fragments.

**Fig 5:**
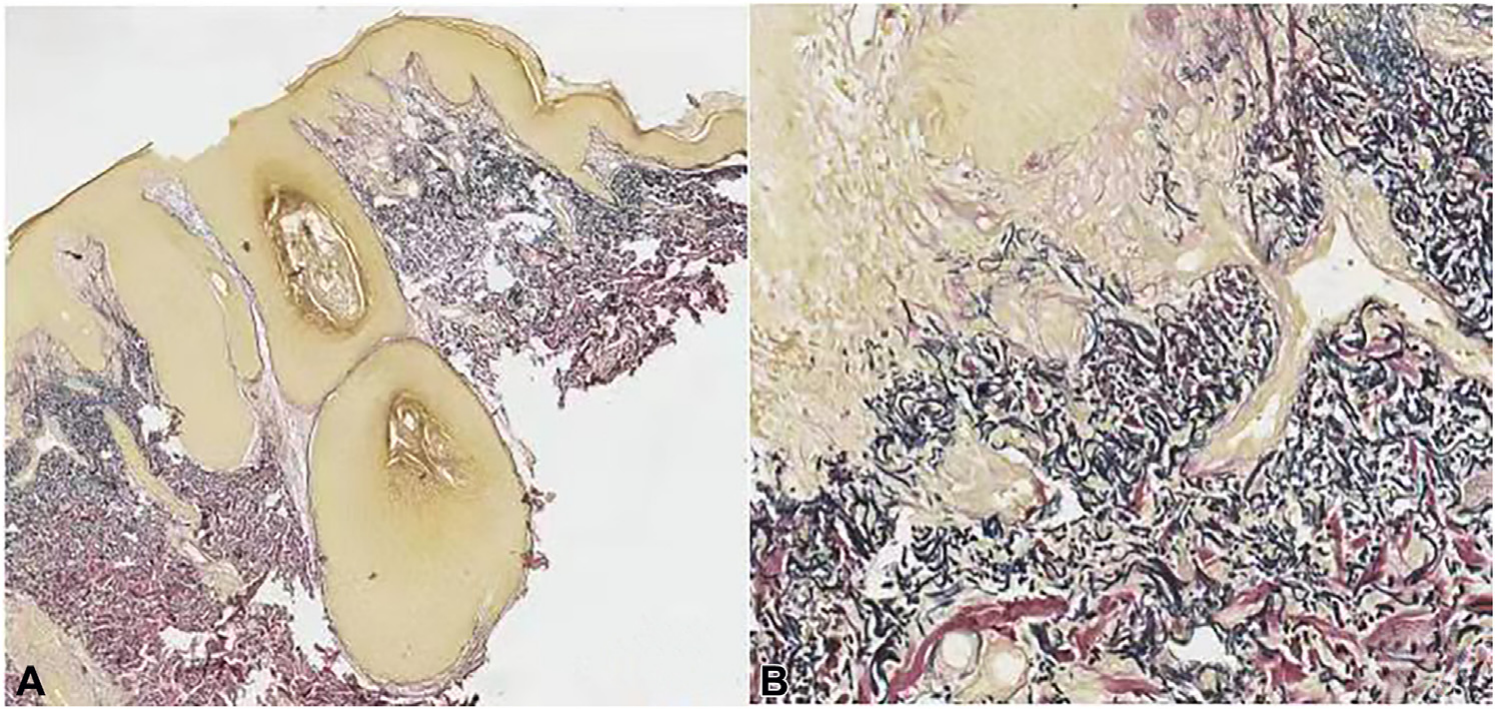
Elastic Van Gieson-stained sections. **A,** Showing accumulation of abnormal elastic fibers in the upper part of the dermis (original magnification ×40 abnormal elastic fibers are *black*). **B,** A partial magnification of (**A**), showing accumulation of abnormal elastic fibers (original magnification ×100).

PXE is a rare disease. Both descriptions of dermoscopy and RCM for PXE are limited. This case described some new findings of EPS-like PXE. Previous study^4^ reported dermoscopy and RCM findings for EPS-like PXE, which are similar with our findings, such as keratotic plugs, predominant yellowish orange color background, pigmentation, and microulcerations in the images of dermoscopy. Hyperreflective material filling the dermal papillae like “eggs in the basket” in RCM scans, the similar characteristic in our case is that “cotton wadding.” Ramírez-Bellver et al^5^ showed yellowish color in the center and keratotic papules were surrounded by structures similar to “chrysalides” in the dermoscopy image. Cristián Navarrete-Dechent et al^6^ revealed a central whitish structureless area with a crown of arborizing vessels. In our case, the multiple keratotic plugs (strawberry seed-like signs) in dermoscopy findings have not been reported before. These are likely related to well-defined, oval, moderate to hyperreflective lumpy material on the epidermis in RCM, and the elimination of calcified altered elastic fibers and basophilic fragments in histology. Yellowish-.orange areas in dermoscopy are probably related to hyperreflective curly amorphous material in the upper dermis in RCM scans. This amorphous material resembles “cotton wadding,” which may relate to the transversal cleavage of calcified elastic fibers in histology, with the former potentially corresponding to more calcified elastic fibers. Microulcerations in dermoscopy likely reflect epithelial disruptions caused by the transepidermal elimination of altered elastic fibers in histology which corresponds to the absence of epidermis and scattered inflammatory cell infiltration in the upper dermis in RCM images. In conclusion, the new findings detected by noninvasive techniques for perforating PXE may reveal some clues which is useful to detect these changes observed at histopathology.

## Conflicts of interest

None disclosed.
